# Supplementary material for: Impact of Hypertension on COVID-19 Burden in Kidney Transplant Recipients: An Observational Cohort Study
Source: Viruses. 2022 Oct 30;14(11):2409. doi: 10.3390/v14112409 (PMC9698847; doi:10.3390/v14112409)
Supplement: Supplementary file 1 [file viruses-14-02409-s001.zip › viruses-1977432-supplementary.pdf]

**Figure S1.** Flowchart of patients included for selection of study patients. DGF = Delayed graft function.

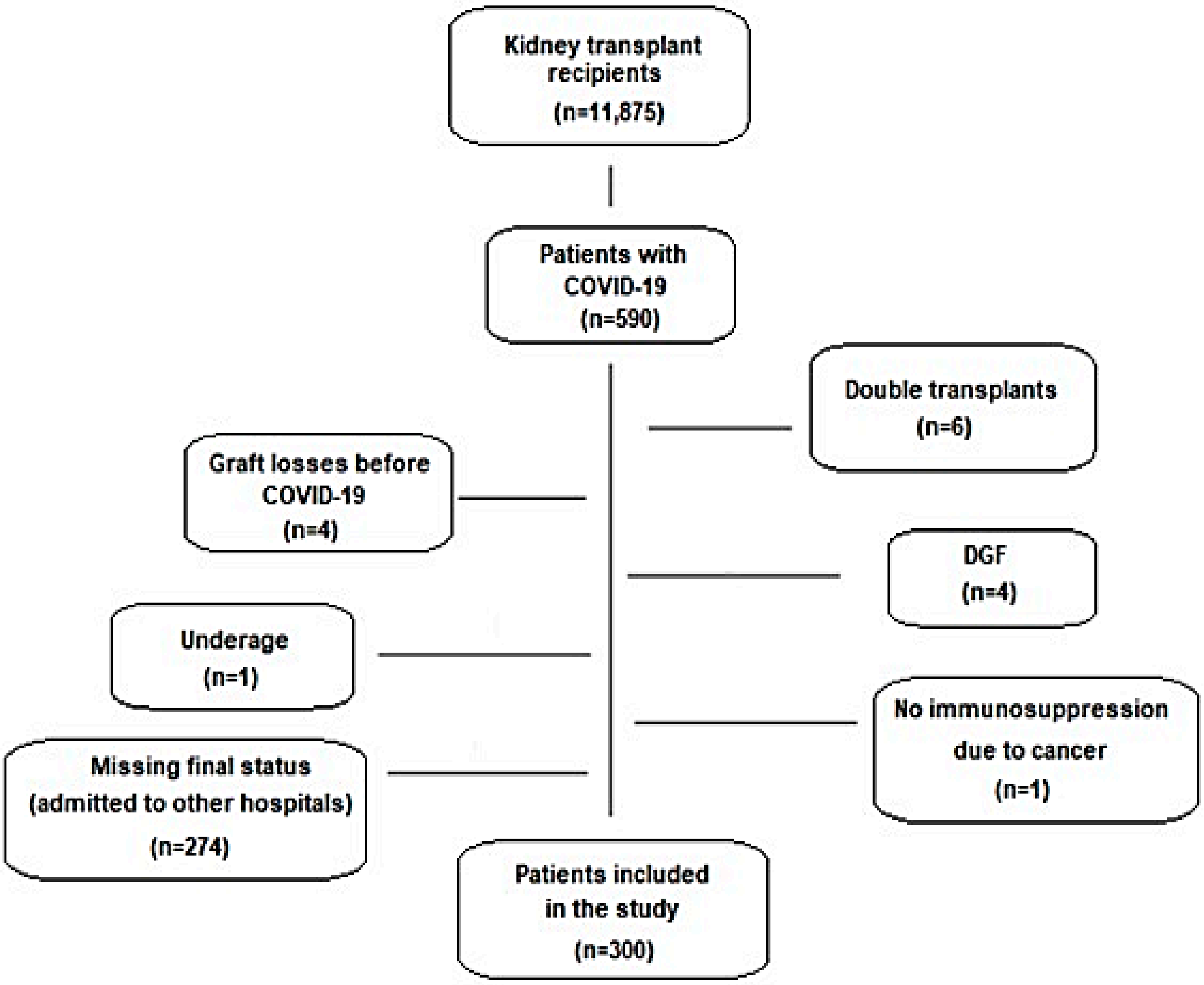

Univariable and multivariable analyses of the outcomes in kidney transplant recipients (**Tables 1S-5S**).

**Table S1.** Risk factors for mortality in kidney transplant recipients.

| | **NOT ALIVE (n=89, 29.7%)** | **ALIVE (n=211, 70.3%)** | **Univariable analysis** | **Multivariable analysis** |
|---|---|---|---|---|
| **Demographic data** | | | | |
| Age (years) | 58.8±10.9 | 49.8±11.7 | 1.07 (1.045-1.097, **p<0.0001**) | 1.05 (1.024-1.084, **p<0.0001**) |
| Male (n, %) | 54 (60.7) | 118 (55.9) | 1.22 (0.734-2.014, p=0.448) | |
| Race (n, %)<br>White<br>Black/brown | 55 (61.8)<br>30 (33.7) | 129 (61.1)<br>80 (37.9) | 1.03 (0.618-1.711, p=0.915) | |
| Transplant time (months) | 85 (41;138) | 71 (31.5;142.5) | 1.00 (0.998-1.005, p=0.493) | |
| Donor type (n, %)<br>Living<br>Deceased | 20 (22.5)<br>69 (77.5) | 65 (30.8)<br>146 (69.2) | 1.54 (0.862-2.736, p=0.145) | |
| BMI (kg/m²) | 27.1±4.7 | 26.8±5.0 | 1.01 (0.958-1.064, p=0.721) | |
| BMI ≥ 25 (n, %) | 38 (42.7) | 82 (38.9) | 1.19 (0.711-1.987, p=0.510) | |
| BMI ≥ 30 (n, %) | 21 (23.6) | 44 (20.9) | 1.19 (0.655-2.159, p=0.569) | |
| HTN (n, %) | 74 (83.1) | 151 (71.6) | 1.96 (1.044-3.682, **p=0.036**) | 1.71 (0.787-3.699, p=0.176) |
| DM (n, %) | 46 (51.7) | 71 (33.6) | 2.11 (1.274-3.493, **p=0.004**) | 1.11 (0.579-2.132, p=0.753) |
| COPD (n, %) | 3 (3.4) | 6 (2.8) | 1.19 (0.291-4.875, p=0.807) | |
| Heart disease (n, %) | 18 (20.2) | 14 (6.6) | 3.57 (1.686-7.547, **p=0.001**) | 3.10 (1.163-8.247, **p=0.024**) |
| Neoplasia (n, %) | 10 (11.2) | 11 (5.2) | 2.30 (0.940-5.633, p=0.068) | 1.95 (0.521-7.281, p=0.322) |
| Liver disease (n, %) | 5 (5.6) | 4 (1.9) | 3.08 (0.807-11.752, p=0.100) | |
| Autoimmune disease (n, %) | 2 (2.2) | 4 (1.9) | 1.19 (0.214-6.616, p=0.843) | |
| Smoking (n, %) | 26 (29.2) | 36 (17.1) | 2.06 (1.126-3.767, **p=0.019**) | 1.87 (0.967-3.596, p=0.063) |
| **Laboratory data** | | | | |
| Basal eGFR | 48.2 (26.5;60.4) | 46.8 (32.2;64.4) | 1.00 (0.986-1.007, p=0.462) | |
| Admission eGFR | 29 (16.9;44.5) | 37.1 (22;52.1) | 0.99 (0.977-1.001, p=0.070) | 1.00 (0.979-1.018, p=0.845) |
| Previous glycemia (mg/dL) | 110.5 (91.5;171.8) | 92 (83;112) | 1.01 (1.004-1.013, **p<0.0001**) | 1.01 (0.999-1.011, p=0.120) |
| CRP (mg/dL) | 12 (5.5;18.8) | 4.4 (1.5;10.1) | 1.08 (1.047-1.121, **p<0.0001**) | 1.03 (0.982-1.090, p=0.205) |
| LDH (U/L) | 353 (277.3;459.8) | 265 (210;351.5) | 1.00 (1.001-1.004, **p=0.004**) | 1.00 (0.999-1.003, p=0.155) |
| Lymphocytes (mm³) | 548 (356.5;836) | 847 (575.3;1212.3) | 1.00 (0.999-1.000, **p=0.013**) | 1.00 (0.998-1.000, p=0.041) |
| D-dimer (µg/L) | 1.4 (0.7;2.7) | 1.1 (0.5;2.0) | 1.13 (1.041-1.215, **p=0.003**) | 1.17 (1.005-1.367, **p=0.043**) |
| AST (U/L) | 34 (21;44.5) | 28 (21;40) | 1.00 (0.996-1.011, p=0.353) | |
| ALT (U/L) | 24 (15;33) | 21 (15;29) | 1.00 (0.991-1.013, p=0.700) | |

BMI: body mass index; HTN: hypertension; DM: diabetes mellitus; COPD: chronic obstructive pulmonary disease; eGFR: estimated glomerular filtration rate (in mL/min/1.73 $m^2$); CRP: C-reactive protein; LDH: lactate dehydrogenase; AST: aspartate aminotransferase; ALT: alanine aminotransferase. Age and BMI are means ± SD, while transplant time, basal eGFR, admission eGFR, previous glycemia, CRP, LDH, lymphocytes, D-dimer, AST, and ALT are medians and IQR. Univariable and multivariable analyses are represented by odds ratios, 95% confidence intervals, and *P*-values, respectively.

**Table S2.** Risk factors for intensive care unit (ICU) admission in kidney transplant recipients.

| | ICU (n=140, 46.7%) | No ICU (n=160, 53.3%) | Univariable analysis | Multivariable analysis |
|---|---|---|---|---|
| **Demographic data** | | | | |
| Age (years) | 56.5±11.7 | 49.0±11.5 | 1.06 (1.035-1.080, **p<0.0001**) | 1.04 (1.013-1.064, **p=0.003**) |
| Male (n, %) | 79 (56.4) | 93 (58.1) | 0.93 (0.590-1.476, p=0.767) | |
| Race (n, %)<br>White<br>Black/brown | 88 (62.9)<br>47 (33.6) | 96 (60.0)<br>63 (39.4) | 1.13 (0.708-1.799, p=0.612) | |
| Transplant time (months) | 84 (35.8;135) | 71.5 (31.8;143.3) | 1.00 (0.997-1.003, p=0.944) | |
| Donor type (n, %)<br>Living<br>Deceased | 40 (28.6)<br>100 (71.4) | 45 (28.1)<br>115 (71.9) | 0.98 (0.591-1.618, p=0.932) | |
| BMI (kg/m²) | 27.3±4.8 | 26.5±4.9 | 1.04 (0.987-1.087, p=0.156) | |
| BMI ≥ 25 (n, %) | 57 (40.7) | 63 (39.4) | 1.02 (0.638-1.638, p=0.927) | |
| BMI ≥ 30 (n, %) | 35 (25.0) | 30 (18.8) | 1.43 (0.818-2.485, p=0.210) | |
| HTN (n, %) | 114 (81.4) | 111 (69.4) | 1.94 (1.125-3.330, **p=0.017**) | 1.69 (0.885-3.222, p=0.112) |
| DM (n, %) | 64 (45.7) | 53 (33.1) | 1.70 (1.065-2.714, **p=0.026**) | 1.03 (0.564-1.876, p=0.928) |
| COPD (n, %) | 6 (4.3) | 3 (1.9) | 2.34 (0.575-9.550, p=0.235) | |
| Heart disease (n, %) | 24 (17.1) | 8 (5.0) | 3.93 (1.704-9.068, **p=0.001**) | 3.55 (1.108-11.378, **p=0.033**) |
| Neoplasia (n, %) | 15 (10.7) | 6 (3.8) | 3.08 (1.161-8.171, **p=0.024**) | 2.90 (0.686-12.237, p=0.148) |
| Liver disease (n, %) | 6 (4.3) | 3 (1.9) | 2.34 (0.575-9.550, p=0.235) | |
| Autoimmune disease (n, %) | 5 (3.6) | 1 (0.6) | 5.89 (0.680-51.025, p=0.108) | |
| Smoking (n, %) | 36 (25.7) | 26 (16.3) | 1.67 (0.933-2.995, p=0.084) | 1.53 (0.820-2.840, p=0.182) |
| **Laboratory data** | | | | |
| Basal eGFR | 45.3 (28.2;60) | 47.9 (32.2;65) | 1.00 (0.985-1.004, p=0.273) | |
| Admission eGFR | 29 (17.1;45) | 39.4 (25.3;52.7) | 0.98 (0.974-0.995, **p=0.005**) | 0.98 (0.958-0.997, **p=0.027**) |
| Previous glycemia (mg/dL) | 105 (89;158.5) | 92 (82;105.5) | 1.01 (1.004-1.014, **p<0.0001**) | 1.00 (0.997-1.010, p=0.278) |
| CRP (mg/dL) | 9.9 (4.2;16.6) | 3.7 (1.2;9.6) | 1.08 (1.039-1.112, **p<0.0001**) | 1.00 (0.938-1.063, p=0.967) |
| LDH (U/L) | 347 (252.5;475) | 247.5 (203.5;308) | 1.01 (1.003-1.007, **p<0.0001**) | 1.01 (1.001-1.010, **p=0.017**) |
| Lymphocytes (mm³) | 585 (377;890.3) | 895 (619;1232) | 1.00 (0.999-1.000, **p=0.020**) | 1.00 (0.998-1.000, **p=0.046**) |
| D-dimer (μg/L) | 1.5 (0.8;2.7) | 0.9 (0.5;1.7) | 1.15 (1.047-1.272, p=0.004) | 1.22 (0.979-1.518, p=0.077) |
| AST (U/L) | 31 (21;42) | 28 (21;39) | 1.01 (0.999-1.018, p=0.082) | 0.99 (0.975-1.010, p=0.378) |
| ALT (U/L) | 21.5 (14;31.8) | 21 (15;31.8) | 1.00 (0.990-1.010, p=0.984) | |

BMI: body mass index; HTN: hypertension; DM: diabetes mellitus; COPD: chronic obstructive pulmonary disease; eGFR: estimated glomerular filtration rate (in mL/min/1.73 $m^2$); CRP: C-reactive protein; LDH: lactate dehydrogenase; AST: aspartate aminotransferase; ALT: alanine aminotransferase. Age and BMI are means ± SD, while transplant time, basal eGFR, admission eGFR, previous glycemia, CRP, LDH, lymphocytes, D-dimer, AST, and ALT are medians and IQR. Univariable and multivariable analyses are represented by odds ratios, 95% confidence intervals, and *P*-values, respectively.

**Table S3.** Risk factors for the need for hemodialysis (HD) admission in kidney transplant recipients.

| | HD (n=109, 36.3%) | No HD (n=191, 63.7%) | Univariable analysis | Multivariable analysis |
|---|---|---|---|---|
| **Demographic data** | | | | |
| Age (years) | 55.6±11.8 | 50.7±12.1 | 1.04 (1.014-1.057, **p=0.001)** | 1.03 (1.004-1.050, **p=0.021**) |
| Male (n, %) | 66 (60.6) | 106 (55.5) | 1.23 (0.763-1.986, p=0.395) | |
| Race (n, %)<br>White<br>Black/brown | 70 (64.2)<br>35 (32.1) | 114 (59.7)<br>75 (39.3) | 1.21 (0.745-1.973, p=0.438) | |
| Transplant time (months) | 85 (35;127) | 73 (32.5;145) | 1.00 (0.996-1.003, p=0.803) | |
| Donor type (n, %)<br>Living<br>Deceased | 24 (22.0)<br>85 (78.0) | 61 (31.9)<br>130 (68.1) | 1.66 (0.963-2.868, p=0.068) | 1.51 (0.847-2.675, p=0.163) |
| BMI (kg/m²) | 27.1±4.6 | 26.8±5.1 | 1.01 (0.965-1.065, p=0.589) | |
| BMI ≥ 25 (n, %) | 43 (39.4) | 77 (40.3) | 0.92 (0.564-1.498, p=0.734) | |
| BMI ≥ 30 (n, %) | 27 (24.8) | 38 (19.9) | 1.29 (0.735-2.277, p=0.372) | |
| HTN (n, %) | 91 (83.5) | 134 (70.2) | 2.15 (1.188-3.891, **p=0.011)** | 1.75 (0.937-3.260, p=0.079) |
| DM (n, %) | 51 (46.8) | 66 (34.6) | 1.67 (1.030-2.692, **p=0.037**) | 1.11 (0.653-1.895, p=0.696) |
| COPD (n, %) | 5 (4.6) | 4 (2.1) | 2.25 (0.591-8.553, p=0.235) | |
| Heart disease (n, %) | 18 (16.5) | 14 (7.3) | 2.50 (1.190-5.256, **p=0.016**) | 1.68 (0.767-3.690, p=0.195) |
| Neoplasia (n, %) | 11 (10.1) | 10 (5.2) | 2.03 (0.834-4.952, p=0.119) | |
| Liver disease (n, %) | 7 (6.4) | 2 (1.0) | 6.49 (1.323-31.796, **p=0.021**) | 4.19 (0.830-21.177, p=0.083) |
| Autoimmune disease (n, %) | 3 (2.8) | 3 (1.6) | 1.77 (0.352-8.943, p=0.488) | |
| Smoking (n, %) | 29 (26.6) | 33 (17.3) | 1.50 (0.835-2.679, p=0.176) | |
| **Laboratory data** | | | | |
| Basal eGFR | 41.5 (21.8;56.7) | 50.6 (35.1;67.3) | 0.98 (0.974-.995, **p=0.003**) | 1.00 (0.981-1.020, p=0.992) |
| Admission eGFR | 23.5 (12.8;40.0) | 40.6 (26;54.1) | 0.97 (0.956-0.981, **p<0.0001**) | 0.98 (0.952-0.999, **p=0.043**) |
| Previous glycemia (mg/dL) | 105 (90;149.5) | 92 (83;111) | 1.01 (1.004-1.013, **p<0.0001**) | 1.00 (0.997-1.009, p=0.323) |
| CRP (mg/dL) | 10.2 (4.3;16.9) | 4.4 (1.4;10.0) | 1.05 (1.021-1.086, **p=0.001**) | 1.00 (0.947-1.048, p=0.882) |
| LDH (U/L) | 351.5 (247.3;488.3) | 265 (211;345.5) | 1.00 (1.001-1.004, **p=0.002**) | 1.00 (1.000-1.004, p=0.071) |
| Lymphocytes (mm³) | 546.5 (377;843) | 864 (597.5;1219.5) | 1.00 (0.999-1.000, p=0.056) | 1.00 (0.997-1.000, **p=0.005**) |
| D-dimer (μg/L) | 1.3 (0.7;2.6) | 1.1 (0.5;2.0) | 1.07 (0.996-1.150, p=0.064) | 1.13 (0.973-1.304, p=0.111) |
| AST (U/L) | 30 (20;42) | 28 (22;40) | 1.00 (0.995-1.009, p=0.553) | |
| ALT (U/L) | 23 (14;32.5) | 21 (15;31) | 1.00 (0.989-1.010, p=0.905) | |

BMI: body mass index; HTN: hypertension; DM: diabetes mellitus; COPD: chronic obstructive pulmonary disease; eGFR: estimated glomerular filtration rate (in mL/min/1.73 $m^2$); CRP: C-reactive protein; LDH: lactate dehydrogenase; AST: aspartate aminotransferase; ALT: alanine aminotransferase. Age and BMI are means ± SD, while transplant time, basal eGFR, admission eGFR, previous glycemia, CRP, LDH, lymphocytes, D-dimer, AST, and ALT are medians and IQR. Univariable and multivariable analyses are represented by odds ratios, 95% confidence intervals, and *P*-values, respectively.

**Table S4.** Risk factors for the use of supplemental oxygen ($O_2$) admission in kidney transplant recipients.

| | **$0_2$ (n=163, 54.3%)** | **No $0_2$ (n=137, 45.7%)** | **Univariable analysis** | **Multivariable analysis** |
|---|---|---|---|---|
| **Demographic data** | | | | |
| Age (years) | 55.5±12.0 | 48.9±11.5 | 1.05 (1.027-1.071, **p<0.0001**) | 1.05 (1.024-1.078, **p<0.0001**) |
| Male (n, %) | 89 (54.6) | 83 (60.6) | 0.78 (0.493-1.241, p=0.297) | |
| Race (n, %) | | | | |
| White | 103 (63.2) | 81 (59.1) | 1.19 (0.745-1.892, p=0.471) | |
| Black/brown | 57 (35.0) | 53 (38.7) | | |
| Transplant time (months) | 73 (38.5;128.5) | 89 (31;148) | 1.00 (0.997-1.003, p=0.848) | |
| Donor type (n, %) | | | | |
| Living | 43 (26.4) | 42 (30.7) | 1.23 (0.746-2.041, p=0.413) | |
| Deceased | 120 (73.6) | 95 (69.3) | | |
| BMI (kg/$m^2$) | 27.5±4.9 | 26.2±4.8 | 1.06 (1.005-1.110, **p=0.030)** | 1.06 (0.968-1.156, p=0.212) |
| BMI ≥ 25 (n, %) | 67 (41.1) | 53 (38.7) | 1.15 (0.714-1.840, p=0.571) | |
| BMI ≥ 30 (n, %) | 41 (25.2) | 24 (17.5) | 1.65 (0.933-2.910, p=0.085) | 1.00 (0.367-2.717, p=0.998) |
| HTN (n, %) | 122 (74.8) | 103 (75.2) | 0.98 (0.581-1.660, p=0.947) | |
| DM (n, %) | 72 (44.2) | 45 (32.8) | 1.62 (1.009-2.593, **p=0.046**) | 0.99 (0.535-1.815, p=0.961) |
| COPD (n, %) | 7 (4.3) | 2 (1.5) | 3.03 (0.619-14.827, p=0.171) | |
| Heart disease (n, %) | 19 (11.7) | 13 (9.5) | 1.26 (0.597-2.652, p=0.545) | |
| Neoplasia (n, %) | 14 (8.6) | 7 (5.1) | 1.75 (0.683-4.455, p=0.244) | |
| Liver disease (n, %) | 8 (4.9) | 1 (0.7) | 7.02 (0.867-56.840, p=0.068) | 3.42 (0.393-29.710, p=0.266) |
| Autoimmune disease (n, %) | 4 (2.5) | 2 (1.5) | 1.70 (0.306-9.415, p=0.545) | |
| Smoking (n, %) | 44 (27.0) | 18 (13.1) | 1.26 (1.216-4.210, **p=0.010)** | 1.86 (0.951-3.626, p=0.070) |
| **Laboratory data** | | | | |
| Basal eGFR | 46.2 (29.4;64.3) | 47.8 (31.8;63.9) | 1.00 (0.987-1.006, p=0.434) | |
| Admission eGFR | 30.4 (17.7;46.4) | 39.3 (22.5;52.5) | 0.99 (0.978-0.998, **p=0.025**) | 0.99 (0.972-1.004, p=0.143) |
| Previous glycemia (mg/dL) | 101 (87;137) | 92 (83;111) | 1.00 (1.000-1.008**,** p=0.070) | 1.00 (0.993-1.004, p=0.700) |
| CRP (mg/dL) | 9.0 (4.1;15.4) | 3.2 (1.1;9.6) | 1.06 (1.028-1.101, **p<0.0001**) | 1.06 (1.002-1.116, **p=0.041**) |
| LDH (U/L) | 337 (241.5;433) | 239 (182.8;303.8) | 1.00 (1.002-1.007, **p<0.0001**) | 1.00 (0.998-1.004, p=0.377) |
| Lymphocytes ($mm^3$) | 653 (409.3;958.5) | 895 (588;1325) | 1.00 (0.999-1.000, **p=0.001**) | 1.00 (0.999-1.000, p=0.092) |
| D-dimer (µg/L) | 1.2 (0.6;2.3) | 1.2 (0.5;2.1) | 1.03 (0.958-1.110, p=0.411) | |
| AST (U/L) | 29.5 (20;43.8) | 28 (22.5;37) | 1.01 (1.000-1.022, p=0.057) | 1.01 (0.972-1.004, p=0.143) |
| ALT (U/L) | 21 (13.8;33) | 21 (16;29.3) | 1.01 (0.996-1.018, p=0.234) | |

BMI: body mass index; HTN: hypertension; DM: diabetes mellitus; COPD: chronic obstructive pulmonary disease; eGFR: estimated glomerular filtration rate (in mL/min/1.73 $m^2$); CRP: C-reactive protein; LDH: lactate dehydrogenase; AST: aspartate aminotransferase; ALT: alanine aminotransferase. Age and BMI are means ± SD, while transplant time, basal eGFR, admission eGFR, previous glycemia, CRP, LDH, lymphocytes, D-dimer, AST, and ALT are medians and IQR. Univariable and multivariable analyses are represented by odds ratios, 95% confidence intervals, and *P*-values, respectively.

**Table S5.** Risk factors for the need for invasive mechanical ventilation (IMV) admission in kidney transplant recipients.

| | **IMV (n=102, 34%)** | **No IMV (n=198, 66%)** | **Univariable analysis** | **Multivariable analysis** |
|---|---|---|---|---|
| **Demographic data** | | | | |
| Age (years) | 57.4±10.6 | 49.9±12.2 | 1.06 (1.034-1.081, **p<0.0001**) | 1.04 (1.014-1.069, **p=0.002**) |
| Male (n, %) | 64 (62.7) | 108 (54.5) | 1.40 (0.860-2.289, p=0.174) | |
| Race (n, %)<br>White<br>Black/brown | 63 (61.8)<br>34 (33.3) | 121 (61.1)<br>76 (38.4) | 1.03 (0.629-1.680, p=0.912) | |
| Transplant time (months) | 84 (38.3;133) | 72.5 (32;143.8) | 1.00 (0.996-1.003, p=0.917) | |
| Donor type (n, %)<br>Living<br>Deceased | 24 (23.5)<br>78 (76.5) | 61 (30.8)<br>137 (69.2) | 1.45 (0.837-2.503, p=0.186) | |
| BMI (kg/m²) | 27.5±4.5 | 26.6±5.0 | 1.04 (0.990-1.094, p=0.121) | |
| BMI ≥ 25 (n, %) | 44 (43.1) | 76 (38.4) | 1.21 (0.739-1.989, p=0.445) | |
| BMI ≥ 30 (n, %) | 26 (25.5) | 39 (19.7) | 1.40 (0.790-2.477, p=0.249) | |
| HTN (n, %) | 83 (81.4) | 142 (71.7) | 1.72 (0.958-3.097, p=0.069) | 1.50 (0.739-3.046, p=0.262) |
| DM (n, %) | 52 (51.0) | 65 (32.8) | 2.13 (1.306-3.469, **p=0.002**) | 1.27 (0.686-2.356, p=0.445) |
| COPD (n, %) | 4 (3.9) | 5 (2.5) | 1.58 (0.414-5.999, p=0.505) | |
| Heart disease (n, %) | 18 (17.6) | 14 (7.1) | 2.82 (1.338-5.930, **p=0.006)** | 2.19 (0.824-5.808, p=0.116) |
| Neoplasia (n, %) | 12 (11.8) | 9 (4.5) | 2.80 (1.138-6.887, **p=0.025)** | 3.54 (0.906-13.856, p=0.069) |
| Liver disease (n, %) | 6 (5.9) | 3 (1.5) | 4.06 (0.995-16.595, p=0.051) | 1.20 (0.232-6.152, p=0.831) |
| Autoimmune disease (n, %) | 2 (2.0) | 4 (2.0) | 0.97 (0.175-5.387, p=0.972) | |
| Smoking (n, %) | 29 (28.4) | 33 (16.7) | 1.86 (1.035-3.357, **p=0.038)** | 1.70 (0.904-3.199, p=0.100) |
| **Laboratory data** | | | | |
| Basal eGFR | 46.2 (28.5;60.3) | 47.2 (31.9;64.5) | 1.00 (0.988-1.008, p=0.708) | |
| Admission eGFR | 29 (17.3;44.3) | 37.5 (21.4;52.4) | 1.00 (0.976-0.999, **p=0.036**) | 1.00 (0.979-1.015, p=0.742) |
| Previous glycemia (mg/dL) | 114 (93;166) | 92 (82.8;108) | 1.01 (1.005-1.014, **p<0.0001**) | 1.01 (0.999-1.012, p=0.082) |
| CRP (mg/dL) | 10.5 (5.0;17.1) | 4.4 (1.5;9.8) | 1.07 (1.031-1.099, **p<0.0001**) | 1.02 (0.973-1.074, p=0.383) |
| LDH (U/L) | 359.5 (266;490.5) | 263 (209;347) | 1.00 (1.001-1.004, **p=0.001**) | 1.00 (1.000-1.003, p=0.114) |
| Lymphocytes (mm³) | 523 (351.5;828.5) | 865 (600.3;1236.3) | 1.00 (0.999-1.000, **p=0.002**) | 1.00 (0.998-1.000, **p=0.015**) |
| D-dimer (µg/L) | 1.3 (0.7;2.6) | 1.1 (0.5;2.0) | 1.09 (1.014-1.175, **p=0.020)** | 1.08 (0.928-1.248, p=0.330) |
| AST (U/L) | 34 (21;42) | 28 (21;40) | 1.00 (0.996-1.010, p=0.455) | |
| ALT (U/L) | 24 (15.5;33) | 21 (15;29) | 1.00 (0.991-1.012, p=0.778) | |

BMI: body mass index; HTN: hypertension; DM: diabetes mellitus; COPD: chronic obstructive pulmonary disease; eGFR: estimated glomerular filtration rate (in mL/min/1.73 $m^2$); CRP: C-reactive protein; LDH: lactate dehydrogenase; AST: aspartate aminotransferase; ALT: alanine aminotransferase. Age and BMI are means ± SD, while transplant time, basal eGFR, admission eGFR, previous glycemia, CRP, LDH, lymphocytes, D-dimer, AST, and ALT are medians and IQR. Univariable and multivariable analyses are represented by odds ratios, 95% confidence intervals, and *P*-values, respectively.

Composite outcomes in kidney transplant recipients (**Tables 6S-7S**).

**Table S6.** Risk factors for composite outcomes of mortality, and ICU and HD in kidney transplant recipients.

| | Composite Outcome (n=78, 26%) | No Composite Outcome (n=150, 50%) | Univariable analysis | Multivariable analysis |
|---|---|---|---|---|
| **Demographic data** | | | | |
| Age (years) | 58.3±10.3 | 48.8±11.4 | 1.06 (1.035-1.086, **p<0.0001**) | 1.05 (1.018-1.079, **p=0.001**) |
| Male (n, %) | 47 (60.3) | 87 (58.0) | 1.18 (0.696-1.990, p=0.544) | |
| Race (n, %) | | | | |
| White | 52 (66.7) | 90 (60.0) | 1.36 (0.793-2.344, p=0.262) | |
| Black/brown | 23 (29.5) | 59 (39.3) | | |
| Transplant time (months) | 84 (41.3;126.8) | 71.5 (32.3;142.8) | 1.00 (0.997-1.004, p=0.898) | |
| Donor type (n, %) | | | | |
| Living | 16 (20.5) | 45 (30.0) | 1.75 (0.941-3.224, p=0.077) | 1.00 (0.441-2.273, p=0.998) |
| Deceased | 62 (79.5) | 105 (70.0) | | |
| BMI (kg/m²) | 27.4±4.4 | 26.7±4.8 | 1.03 (0.974-1.084, p=0.326) | |
| BMI ≥ 25 (n, %) | 34 (43.6) | 61 (40.7) | 1.19 (0.697-2.018, p=0.529) | |
| BMI ≥ 30 (n, %) | 20 (25.6) | 29 (19.3) | 1.33 (0.725-2.451, p=0.354) | |
| HTN (n, %) | 66 (84.6) | 103 (68.7) | 2.18 (1.103-4.305, **p=0.025**) | 2.01 (0.885-4.550, p=0.096) |
| DM (n, %) | 39 (50.0) | 47 (31.3) | 1.85 (1.095-3.113, **p=0.021**) | 1.01 (0.517-1.987, p=0.969) |
| COPD (n, %) | 3 (3.8) | 3 (2.0) | 1.44 (0.351-5.902, p=0.612) | |
| Heart disease (n, %) | 17 (21.8) | 8 (5.3) | 3.85 (1.815-8.148, **p<0.0001**) | 2.94 (1.121-7.732, **p=0.028**) |
| Neoplasia (n, %) | 10 (12.8) | 6 (4.0) | 2.82 (1.148-6.931, **p=0.024**) | 2.38 (0.623-9.053, p=0.205) |
| Liver disease (n, %) | 5 (6.4) | 1 (0.7) | 3.73 (0.976-14.274, p=0.054) | 0.98 (0.190-5.053, p=0.981) |
| Autoimmune disease (n, %) | 2 (2.6) | 0 (0) | 1.43 (0.258-7.988, p=0.681) | |
| Smoking (n, %) | 24 (30.8) | 25 (16.7) | 2.03 (1.096-3.749, **p=0.024**) | 1.86 (0.956-3.630, p=0.067) |
| **Laboratory data** | | | | |
| Basal eGFR | 46.5 (25.2;59.5) | 50.1 (35.4;66.9) | 1.00 (0.984-1.006, p=0.332) | |
| Admission eGFR | 28.2 (16.8;44.3) | 41.3 (26.8;54.7) | 0.99 (0.976-1.001, p=0.061) | 1.00 (0.984-1.024, p=0.713) |
| Previous glycemia (mg/dL) | 110 (92;191) | 92 (82;105) | 1.01 (1.005-1.013, **p<0.0001**) | 1.01 (0.999-1.012, p=0.080) |
| CRP (mg/dL) | 12 (5.9;18.3) | 3.7 (1.2;9.5) | 1.08 (1.040-1.114, **p<0.0001**) | 1.04 (0.985-1.096, p=0.158) |
| LDH (U/L) | 360.5 (282.3;459.8) | 250 (201;309.5) | 1.00 (1.000-1.004, **p=0.010**) | 1.00 (0.999-1.003, p=0.391) |
| Lymphocytes (mm³) | 534.5 (352.3;818.8) | 877 (632;1206) | 1.00 (0.999-1.000, **p=0.009**) | 1.00 (0.997-1.000, **p=0.024**) |
| D-dimer (µg/L) | 1.5 (0.8;2.7) | 0.9 (0.5;1.7) | 1.09 (1.017-1.176, **p=0.016**) | 1.15 (0.983-1.338, p=0.081) |
| AST (U/L) | 33 (21;43.3) | 28 (21.3;38.8) | 1.00 (0.996-1.011, p=0.325) | |
| ALT (U/L) | 24 (15;33) | 21 (15;32.5) | 1.00 (0.992-1.014, p=0.575) | |

BMI: body mass index; HTN: hypertension; DM: diabetes mellitus; COPD: chronic obstructive pulmonary disease; eGFR: estimated glomerular filtration rate (in mL/min/1.73 m$^2$); CRP: C-reactive protein; LDH: lactate dehydrogenase; AST: aspartate aminotransferase; ALT: alanine aminotransferase. Age and BMI are means ± SD, while transplant time, basal eGFR, admission eGFR, previous glycemia, CRP, LDH, lymphocytes, D-dimer, AST, and ALT are medians and IQR. Univariable and multivariable analyses are represented by odds ratios, 95% confidence intervals, and *P*-values, respectively.

**Table S7.** Risk factors for composite outcomes of mortality, or ICU or HD in kidney transplant recipients.

| | **Composite Outcome (n=150, 50%)** | **No Composite Outcome (n=150, 50%)** | **Univariable analysis** | **Multivariable analysis** |
|---|---|---|---|---|
| **Demographic data** | | | | |
| Age (years) | 56.1±11.9 | 48.8±11.4 | 1.06 (1.033-1.077, **p<0.0001**) | 1.04 (1.020-1.067, **p<0.0001**) |
| Male (n, %) | 85 (56.7) | 87 (58.0) | 0.95 (0.599-1.496, p=0.815) | |
| Race (n, %)<br>White<br>Black/brown | <br>94 (62.7)<br>51 (34.0) | <br>90 (60.0)<br>59 (39.3) | 1.12 (0.703-1.781, p=0.635) | |
| Transplant time (months) | 84 (34.3;137) | 71.5 (32.3;142.8) | 1.00 (0.997-1.003, p=0.957) | |
| Donor type (n, %)<br>Living<br>Deceased | <br>40 (26.7)<br>110 (73.3) | <br>45 (30.0)<br>105 (70.0) | 1.18 (0.713-1.949, p=0.522) | |
| BMI ($kg/m^2$) | 27.1±4.9 | 26.7±4.8 | 1.02 (0.970-1.068, p=0.467) | |
| BMI ≥ 25 (n, %) | 59 (39.3) | 61 (40.7) | 0.92 (0.575-1.475, p=0.733) | |
| BMI ≥ 30 (n, %) | 36 (24.0) | 29 (19.3) | 1.31 (0.752-2.286, p=0.340) | |
| HTN (n, %) | 122 (81.3) | 103 (68.7) | 1.99 (1.163-3.399, **p=0.012**) | 1.62 (0.909-2.898, p=0.102) |
| DM (n, %) | 70 (46.7) | 47 (31.3) | 1.92 (1.197-3.071, **p=0.007**) | 1.25 (0.739-2.125, p=0.403) |
| COPD (n, %) | 6 (4.0) | 3 (2.0) | 2.04 (0.501-8.319, p=0.319) | |
| Heart disease (n, %) | 24 (16.0) | 8 (5.3) | 3.38 (1.466-7.795, **p=0.004**) | 1.84 (0.759-4.476, p=0.177) |
| Neoplasia (n, %) | 15 (10.0) | 6 (4.0) | 2.67 (1.005-7.073, **p=0.049**) | 1.59 (0.534-4.710, p=0.406) |
| Liver disease (n, %) | 8 (5.3) | 1 (0.7) | 8.39 (1.037-67.971, **p=0.046**) | 4.46 (0.520-38.257, p=0.173) |
| Autoimmune disease (n, %) | 6 (4.0) | 0 (0) | - (-, p=0.999) | |
| Smoking (n, %) | 37 (24.7) | 25 (16.7) | 1.53 (0.852-2.747, p=0.154) | |
| **Laboratory data** | | | | |
| Basal eGFR | 43 (25.1;58.7) | 50.1 (35.4;66.9) | 0.99 (0.980-0.999, **p=0.026**) | 1.00 (0.978-1.024, p=0.945) |
| Admission eGFR | 26 (14.9;44.3) | 41.3 (26.8;54.7) | 0.98 (0.966-0.988 **p<0.0001**) | 0.97 (0.942-0.995, **p=0.019**) |
| Previous glycemia (mg/dL) | 104 (89;148.8) | 92 (82;105) | 1.01 (1.004-1.013, **p=0.001**) | 1.00 (0.995-1.008, p=0.566) |
| CRP (mg/dL) | 9.7 (3.8;16.9) | 3.7 (1.2;9.5) | 1.08 (1.044-1.121, **p<0.0001**) | 1.01 (0.947-1.079, p=0.749) |
| LDH (U/L) | 345 (238;466.8) | 250 (201;309.5) | 1.01 (1.003-1.007, **p<0.0001**) | 1.01 (1.000-1.009, **p=0.035**) |
| Lymphocytes ($mm^3$) | 594 (391.8;958.5) | 877 (632;1206) | 1.00 (0.999-1.000, p=0.051) | 1.00 (0.999-1.000, p=0.111) |
| D-dimer (μg/L) | 1.5 (0.7;2.7) | 0.9 (0.5;1.7) | 1.17 (1.050-1.297, **p=0.004**) | 1.41 (1.043-1.918, **p=0.026**) |
| AST (U/L) | 31 (20.5;42) | 28 (21.3;38.8) | 1.01 (0.999-1.018, p=0.093) | 0.99 (0.976-1.012, p=0.487) |
| ALT (U/L) | 22 (14;31) | 21 (15;32.5) | 1.00 (0.989-1.009, p=0.814) | |

BMI: body mass index; HTN: hypertension; DM: diabetes mellitus; COPD: chronic obstructive pulmonary disease; eGFR: estimated glomerular filtration rate (in mL/min/1.73 $m^2$); CRP: C-reactive protein; LDH: lactate dehydrogenase; AST: aspartate aminotransferase; ALT: alanine aminotransferase. Age and BMI are means ± SD, while transplant time, basal eGFR, admission eGFR, previous glycemia, CRP, LDH, lymphocytes, D-dimer, AST, and ALT are medians and IQR. Univariable and multivariable analyses are represented by odds ratios, 95% confidence intervals, and *P*-values, respectively.

Risk factors for mortality, ICU admission, and HD requirement in kidney transplant recipients without hypertension (HTN).

**Table S8.** Risk factors for mortality in kidney transplant recipients without hypertension (HTN).

| | **NOT ALIVE (n=15, 20%)** | **ALIVE (n=60, 80%)** | **Univariable Analysis** | **Multivariable Analysis** |
|---|---|---|---|---|
| **Demographic Data** | | | | |
| Age (years) | 59.8±13.3 | 48.0±13.3 | 1.07 (1.018-1.116, **p=0.006**) | 1.05 (1.006-1.104, **p=0.026**) |
| Male (n, %) | 10 (66.7) | 26 (43.3) | 2.62 (0.797-8.586, p=0.113) | |
| Race (n, %) | | | | |
| White | 9 (60.0) | 41 (68.3) | 1.30 (0.689-2.451, p=0.418) | |
| Black/brown | 6 (40.0) | 19 (31.7) | | |
| Transplant time (months) | 109 (69;176) | 92 (37.5;139.8) | 1.00 (0.996-1.012, p=0.370) | |
| Donor type (n, %) | | | | |
| Living | 6 (40.0) | 21 (35.0) | 0.81 (0.253-2.580, p=0.718) | |
| Deceased | 9 (60.0) | 39 (65.0) | | |
| BMI (kg/m²) | 26.3±5.9 | 26.0±5.2 | 1.01 (0.906-1.125, p=0.866) | |
| BMI ≥ 25 (n, %) | 6 (40.0) | 21 (35.0) | 1.05 (0.3253.372-, p=0.938) | |
| BMI ≥ 30 (n, %) | 4 (26.7) | 9 (15.0) | 1.90 (0.482-7.163, p=0.368) | |
| DM (n, %) | 6 (40.0) | 11 (18.3) | 2.97 (0.874-10.085, p=0.081) | 2.33 (0.600-9.054, p=0.221) |
| COPD (n, %) | 1 (6.7) | 1 (1.7) | 4.21 (0.248-71.584, p=0.320) | |
| Heart disease (n, %) | 1 (6.7) | 1 (1.7) | 4.21 (0.248-71.584, p=0.320) | |
| Neoplasia (n, %) | 3 (20.0) | 3 (5.0) | 4.75 (0.853-26.450, p=0.075) | 4.01 (0.614-26.213, p=0.147) |
| Autoimmune disease (n, %) | 1 (6.7) | 1 (1.7) | 4.21 (0.248-71.584, p=0.320) | |
| Smoking (n, %) | 4 (26.7) | 10 (16.7) | 1.73 (0.442-6.804, p=0.430) | |
| **Laboratory Data** | | | | |
| Basal eGFR | 36 (28;54) | 51 (35;66) | 0.98 (0.948-1.006, p=0.119) | |
| Admission eGFR | 24 (19;34) | 41 (22;58) | 0.96 (0.930-0.995, **p=0.023**) | 0.97 (0.924-1.018, p=0.217) |
| Previous glycemia (mg/dL) | 104 (85;127.5) | 92.5 (80.8;104.3) | 1.00 (0.994-1.008, p=0.777) | |
| CRP (mg/dL) | 10.5 (7.3;15.5) | 5.0 (0.7;8.8) | 1.10 (1.015-1.201, **p=0.021**) | 0.96 (0.816-1.132, p=0.637) |
| LDH (U/L) | 341 (293.5;390.5) | 241.5 (178.5;322.5) | 1.01 (1.001-1.009, **p=0.019**) | 1.01 (0.996-1.024, p=0.169) |
| Lymphocytes (mm³) | 700.5 (410;890.3) | 895 (601;1176.5) | 1.00 (0.997-1.000, p=0.112) | |
| D-dimer (μg/L) | 1.3 (0.9;2.5) | 0.9 (0.5;1.6) | 1.31 (0.957-1.793, p=0.092) | 1.33 (0.851-2.079, p=0.210) |
| AST (U/L) | 37 (32;69.3) | 29 (22.8;40.8) | 1.02 (0.999-1.043, p=0.066) | 1.00 (0.959-1.035, p=0.839) |
| ALT (U/L) | 26 (24;33) | 19 (15.8;27.3) | 1.01 (0.980-1.031, p=0.688) | |

HTN: hypertension; BMI: body mass index; COPD: chronic obstructive pulmonary disease; eGFR: estimated glomerular filtration rate (in mL/min/1.73 m$^2$); CRP: C-reactive protein; LDH: lactate dehydrogenase; AST: aspartate aminotransferase; ALT: alanine aminotransferase. Age and BMI are means ± SD, while transplant time, basal eGFR, admission eGFR, previous glycemia, CRP, LDH, lymphocytes, D-dimer, AST, and ALT are medians and IQR. Univariable and multivariable analyses are represented by odds ratios, 95% confidence intervals, and *P*-values, respectively.

**Table S9.** Risk factors for intensive care unit (ICU) admission in kidney transplant recipients without hypertension (HTN).

| | **ICU (n=26, 34.7%)** | **NO ICU (n=49, 65.3%)** | **Univariable Analysis** | **Multivariable Analysis** |
|---|---|---|---|---|
| **Demographic Data** | | | | |
| Age (years) | 54.5±14.0 | 48.2±13.7 | 1.03 (0.998-1.071, p=0.067) | 1.02 (0.987-1.063, p=0.201) |
| Male (n, %) | 14 (53.8) | 22 (44.9) | 1.43 (0.551-3.720, p=0.461) | |
| Race (n, %) | | | | |
| White | 18 (69.2) | 32 (65.3) | 1.01 (0.577-1.761, p=0.979) | |
| Black/brown | 8 (30.8) | 17 (34.7) | | |
| Transplant time (months) | 81.5 (30.8;129.3) | 106 (46;148) | 1.00 (0.990-1.004, p=0.442) | |
| Donor type (n, %) | | | | |
| Living | 11 (42.3) | 16 (32.7) | 0.66 (0.248-1.763, p=0.408) | |
| Deceased | 15 (57.7) | 33 (67.3) | | |
| BMI (kg/m²) | 26.9±5.7 | 25.5±5.0 | 1.05 (0.957-1.156, p=0.295) | |
| BMI ≥ 25 (n, %) | 9 (34.6) | 18 (36.7) | 0.81 (0.295-2.240, p=0.688) | |
| BMI ≥ 30 (n, %) | 6 (23.1) | 7 (14.3) | 1.71 (0.505-5.816, p=0.387) | |
| DM (n, %) | 11 (42.3) | 6 (12.2) | 5.26 (1.655-16.688, **p=0.005**) | 4.55 (1.392-14.847, **p=0.012**) |
| COPD (n, %) | 1 (3.8) | 1 (2.0) | 1.92 (0.115-32.008, p=0.650) | |
| Heart disease (n, %) | 1 (3.8) | 1 (2.0) | 1.92 (0.115-32.008, p=0.650)) | |
| Neoplasia (n, %) | 4 (15.4) | 2 (4.1) | 4.27 (0.727-25.117, p=0.108) | |
| Autoimmune disease (n, %) | 2 (7.7) | 0 (0) | - (-, p=0.999) | |
| Smoking (n, %) | 5 (19.2) | 9 (18.4) | 0.93 (0.268-3.198, p=0.903) | |
| **Laboratory Data** | | | | |
| Basal eGFR | 47 (35;56) | 49 (32;66) | 0.99 (0.971-1.017, p=0.591) | |
| Admission eGFR | 29 (21;41) | 42 (22;57) | 0.98 (0.961-1.006, p=0.146) | |
| Previous glycemia (mg/dL) | 102.5 (87.8;131.8) | 89 (80;103) | 1.00 (0.996-1.009, p=0.505) | |
| CRP (mg/dL) | 8.8 (5.1;12.2) | 3.7 (0.4;9.1) | 1.05 (0.980-1.130, p=0.159) | |
| LDH (U/L) | 341 (275.5;412) | 225.5 (175.8;298.8) | 1.01 (1.002-1.012, **p=0.004**) | 1.01 (1.001-1.011, **p=0.012**) |
| Lymphocytes (mm³) | 548.5 (378.8;864.8) | 931 (729;1206) | 1.00 (0.997-1.000, **p=0.017**) | 1.00 (0.998-1.000, p=0.137) |
| D-dimer (µg/L) | 1.3 (0.7;2.0) | 0.9 (0.5;1.6) | 1.05 (0.812-1.358, p=0.710) | |
| AST (U/L) | 36 (26;63) | 30 (23;43) | 1.01 (0.993-1.030, p=0.238) | |
| ALT (U/L) | 20 (16.3;31.5) | 21 (16;28) | 0.99 (0.964-1.018, p=0.502) | |

HTN: hypertension; BMI: body mass index; COPD: chronic obstructive pulmonary disease; eGFR: estimated glomerular filtration rate (in mL/min/1.73 $m^2$); CRP: C-reactive protein; LDH: lactate dehydrogenase; AST: aspartate aminotransferase; ALT: alanine aminotransferase. Age and BMI are means ± SD, while transplant time, basal eGFR, admission eGFR, previous glycemia, CRP, LDH, lymphocytes, D-dimer, AST, and ALT are medians and IQR. Univariable and multivariable analyses are represented by odds ratios, 95% confidence intervals, and *P*-values, respectively.

**Table S10.** Risk factors for the need for hemodialysis (HD) in kidney transplant recipients without hypertension (HTN).

| | **HD (n=18, 24.0%)** | **NO HD (n=57, 76.0%)** | **Univariable Analysis** | **Multivariable Analysis** |
|---|---|---|---|---|
| **Demographic Data** | | | | |
| Age (years) | 51.5±14.9 | 50.0±13.9 | 1.01 (0.970-1.046, p=0.691) | |
| Male (n, %) | 10 (55.6) | 26 (45.6) | 1.49 (0.513-3.327, p=0.463) | |
| Race (n, %) | | | | |
| White | 13 (72.2) | 37 (64.9) | 0.87 (0.458-1.658, p=0.676) | |
| Black/brown | 5 (27.8) | 20 (35.1) | | |
| Transplant time (months) | 77.5 (24.3;126.8) | 105 (46;148) | 1.00 (0.987-1.003, p=0.242) | |
| Donor type (n, %) | | | | |
| Living | 7 (38.9) | 20 (35.1) | 0.85 (0.285-2.533, p=0.770) | |
| Deceased | 11 (61.1) | 37 (64.9) | | |
| BMI (kg/m²) | 26.3±5.0 | 26.0±5.4 | 1.01 (0.916-1.123, p=0.789) | |
| BMI ≥ 25 (n, %) | 6 (33.3) | 21 (36.8) | 0.71 (0.231-2.206, p=0.559) | |
| BMI ≥ 30 (n, %) | 4 (22.2) | 9 (15.8) | 1.37 (0.363-5.127, p=0.645) | |
| DM (n, %) | 6 (33.3) | 11 (19.3) | 2.09 (0.642-6.807, p=0.221) | |
| COPD (n, %) | 1 (5.6) | 1 (1.8) | 3.29 (0.195-55.511, p=0.408) | |
| Heart disease (n, %) | 1 (5.6) | 1 (1.8) | 3.29 (0.195-55.511, p=0.408) | |
| Neoplasia (n, %) | 3 (16.7) | 3 (5.3) | 3.60 (0.658-19.696, p=0.140) | |
| Autoimmune disease (n, %) | 1 (5.6) | 1 (1.8) | 3.29 (0.195-55.511, p=0.408) | |
| Smoking (n, %) | 3 (16.7) | 11 (19.3) | 0.66 (0.160-2.741, p=0.570) | |
| **Laboratory Data** | | | | |
| Basal eGFR | 43 (30;52) | 52 (34;66) | 0.98 (0.953-1.007, p=0.141) | |
| Admission eGFR | 23 (10;29) | 43 (26;59) | 0.94(0.901-0.975, **p=0.001**) | 0.94 (0.898-0.987, **p=0.012**) |
| Previous glycemia (mg/dL) | 102.5(93.8;117.3) | 90 (80;103) | 1.00 (0.995-1.008, p=0.700) | |
| CRP (mg/dL) | 10.4 (4.3;12.8) | 5.1 (0.5;9.5) | 1.05 (0.973-1.132, p=0.208) | |
| LDH (U/L) | 341.5 (256.8;457.3) | 263 (180;327) | 1.01 (1.001-1.010, **p=0.009**) | 1.01 (1.001-1.011, **p=0.020**) |
| Lymphocytes (mm³) | 597.5 (391.8;864.8) | 918 (632;1206) | 1.00 (0.997-1.000, p=0.051) | 1.00 (0.997-1.001, p=0.222) |
| D-dimer (µg/L) | 1.2 (0.9;1.8) | 0.9 (0.5;1.8) | 0.95 (0.679-1.319, p=0.745) | |
| AST (U/L) | 34 (24;68) | 31 (23.5;43) | 1.01 (0.994-1.030, p=0.118) | |
| ALT (U/L) | 25 (16.5;31.5) | 20 (16;28) | 1.00 (0.967-1.026, p=0.793) | |

HTN: hypertension; BMI: body mass index; COPD: chronic obstructive pulmonary disease; eGFR: estimated glomerular filtration rate (in mL/min/1.73 m2); CRP: C-reactive protein; LDH: lactate dehydrogenase; AST: aspartate aminotransferase; ALT: alanine aminotransferase. Age and BMI are means ± SD, while transplant time, basal eGFR, admission eGFR, previous glycemia, CRP, LDH, lymphocytes, D-dimer, AST, and ALT are medians and IQR. Univariable and multivariable analyses are represented by odds ratios, 95% confidence intervals, and *P*-values, respectively.
